# Accurate Determination of the Temperature Sensitivity of UV-Induced Fiber Bragg Gratings

**DOI:** 10.3390/s26020435

**Published:** 2026-01-09

**Authors:** Miguel Cosme, Marizane Pota, João Preizal, Paulo Caldas, Ricardo Oliveira, Rogério Nogueira, Francisco M. Araújo, José L. Cruz, Gaspar M. Rego

**Affiliations:** 1 HBK FiberSensing, S. A., 4485-860 Maia, Portugal; miguel.cosme@hbkworld.com (M.C.); francisco.araujo@hbkworld.com (F.M.A.); 2 Applied Digital Transformation Laboratory (ADiT-LAB), Instituto Politécnico de Viana do Castelo, Rua Escola Industrial e Comercial Nun’Álvares, 4900-347 Viana do Castelo, Portugal; marizanepacamutondo@ipvc.pt; 3 Instituto de Telecomunicações, University of Aveiro, Campus Universitário de Santiago, 3810-193 Aveiro, Portugal; joaopreizal@ua.pt (J.P.); oliveiraricas@av.it.pt (R.O.); rnogueira@av.it.pt (R.N.); 4 Center for Applied Photonics, Institute for Systems and Computer Engineering, Technology and Science (INESC TEC), Rua Dr. Roberto Frias, 4200-465 Porto, Portugal; pcaldas@estg.ipvc.pt; 5 Center for Research and Development in Agrifood Systems and Sustainability (CISAS), Escola Superior de Tecnologia e Gestão, Instituto Politécnico de Viana do Castelo, 4900-347 Viana do Castelo, Portugal; 6 Department of Applied Physics, Universidad de Valencia, Dr. Moliner 50, 46100 Burjassot, Spain; jose.l.cruz@uv.es

**Keywords:** fiber Bragg gratings, temperature measurement calibration, normalized temperature sensitivity, thermo-optic coefficient

## Abstract

Over the past 18 months, we have performed hundreds of temperature characterizations of fiber Bragg gratings inscribed in different germanium-doped silica glass fibers. Under experimental conditions, the main conclusions are as follows: the temperature dependence of the “temperature gauge factor” or the normalized temperature sensitivity, K*_T_*, was found to be quadratic in the −50–200 °C range, while it may be considered linear for the −20–100 °C range; K*_T_* values at 20 °C vary from 5.176 × 10^−6^ K^−1^, for a B/Ge co-doped fiber up to 6.724 × 10^−6^ K^−1^, for a highly Ge-doped fiber; K*_T_* does not depend on the hydrogen-loading process or the gratings coupling strength; K*_T_* is essentially independent of wavelength in the 1500–1600 nm range, its value being accurately determined with a relative error ~0.2%; based on the accurate value of K*_T_* = 6.165 × 10^−6^ K^−1^, at 20 °C, obtained for gratings inscribed in the SMF-28 fiber, we calculated a value of 19.4 × 10^−6^ K^−1^ for the thermo-optic coefficient of bulk germanium glass; and gratings produced by femtosecond-laser radiation and UV-laser radiation exhibit comparable values of K*_T_*. The previous achievements allow, by having knowledge of K*_T_* for a single grating, the accurate determination of the temperature dependence of the Bragg wavelength for any other grating inscribed in the same fiber; the presented methodology enables one to determine the “unknown” gratings’ temperature sensitivity, typically with an error of 0.01 pm/°C, being, therefore, very useful in research labs and computer simulations. Thus, expressions for the temperature dependence of K*_T_* for gratings inscribed in several fibers are given, as well as an expression for K*_T_* as a function of the effective refractive index. We have also fully analyzed the potential sources of error in K*_T_* determination.

## 1. Introduction

For more than three decades, temperature sensing has probably been the most well-known application of fiber Bragg gratings (FBG) [1,2,3,4,5,6]. Independently of the physical parameter under measurement, temperature discrimination is required [7,8,9,10,11,12,13]. In particular, the development of optical fiber sensors for harsh environments [14], such as high temperature [15,16] and cryogenic applications [17,18,19], is a demanding task. In the latter context and based on our experience with arc-induced gratings [20], we recently discussed their use as high-sensitivity cryogenic temperature sensors [18]. However, some limitations in the presented model were pointed out, mainly concerning the lack of accurate values for fiber parameters, such as the temperature dependence of the glass thermo-optic coefficients. Therefore, in recent years, we have been gathering accurate data on that topic. First, on synthetic fused silica glass [21], based on the work of Leviton and colleagues [22], and then on germanium-doped silica glass [23] relying on the temperature behavior of FBGs, where the requirement for more precise measurements was identified. Thus, in comparison to the number of publications related to FBG theory, applications and simulations, only a few acknowledge the fact that the Bragg wavelength shifts nonlinearly with temperature, even for a limited temperature range, such as 120 °C, and that the thermo-optic coefficient of silica-doped glass fibers depends on temperature and wavelength [21,23,24,25,26]. Recently [25], preliminary results pointed toward the independence of wavelength of the “temperature gauge factor” K*_T_* = (1/*λ*)(d*λ*/dT) [3,27] or the normalized temperature sensitivity, in the 1500–1600 nm range. However, we recognize that definite conclusions require higher stability, accuracy and resolution of the equipment used. Therefore, in this paper, we compare the normalized temperature sensitivity of fs-FBGs and UV-FBGs, and we discuss the potential effect of the hydrogen-loading process and of the initial grating coupling strength on K*_T_* values. We demonstrate that K*_T_* is, essentially, independent of wavelength in the 1500–1600 nm wavelength range, even for highly germanium-doped silica glass fibers. The K*_T_* expressions for FBGs inscribed in silica fibers with distinct concentrations of GeO_2_ are also given. Based on the previous results, we also demonstrate that the temperature dependence of the Bragg wavelength of a second FBG can be accurately predicted through the integration of the K*_T_* expression obtained previously for a former FBG, in an approach that resembles recently published work [28,29]. A thorough discussion of the experimental errors that can affect the determination of the FBG temperature sensitivity is also presented.

## 2. Gratings Fabrication

**Figure 1:**
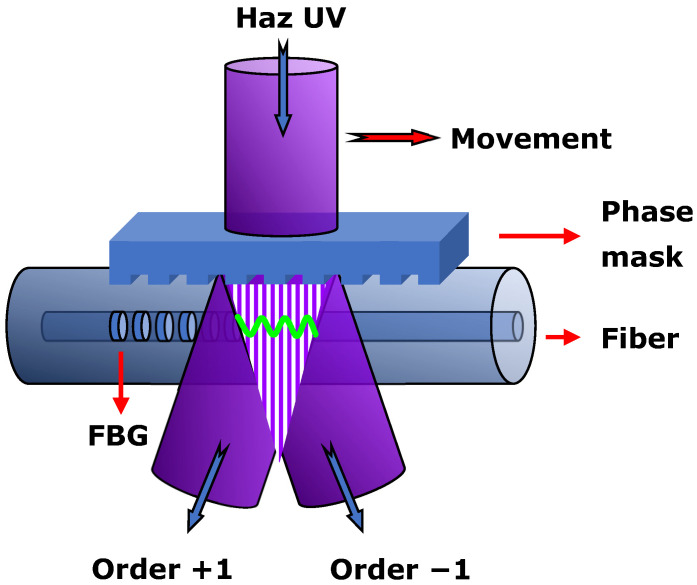
Schematic of the fiber Bragg grating recording system based on the phase mask method.

The UV-FBGs were inscribed in six different fibers, their properties being presented in Table 1. Gratings were made by scanning a doubled argon laser UV-laser (*λ*laser = 244 nm, nominal power = 100 mW, laser beam cross section = 0.9 × 0.9 mm^2^) over the fibers through a phase mask Ibsen Photonics^®^ (period = 1063.50 ± 0.01 nm and length = 10 mm), as depicted in Figure 1. Different grating strengths were achieved by changing several parameters. The fluence on the fiber was controlled by reducing the beam power, by focusing the beam by a cylindric lens if necessary and, mainly, by accurate control of the scan speed. The fluence was reduced so that the gratings had an index change below 10^−5^ (1 cm long gratings with reflectivity below 1%) [30]. The reflectivity was measured taking as reference the reflection from a fiber tip cut at 0°. For the specific gratings produced (PS1250/1500 and SM1500 fibers), the laser power had been in the range 10 mW to 70 mW, and the beam scanning speed in the range 0.01685 mm/s to 1.348 mm/s. Gratings in the PS 1250/1500 fiber and grating of 33% reflectivity (R) in the SM1500 fiber were made without a focusing lens. Gratings with R = 12%, 2.8% and 0.7% in SM1500 fiber (NA = 0.30) were made with a divergent cylindric lens (intensity in the fiber decreased by a factor of 10), and the phase mask period was 1056.50 nm (Table 1). The estimated fluence, F (J/mm^2^), is also given in Table 2. It should be stressed that the choice of the phase mask period, *Λ*, was such that the Bragg wavelength (*λ*_B_ = *n*_eff_*Λ*, *n*_eff_ being the effective refractive index) would be in the 1500–1600 nm range. For instance, for the SMF-28 fiber, a *λ*_B_ ~1539 nm @20 °C was measured (used in the thermal characterization in the next section). Note also that the exact value depends on the fiber strain during inscription and on the grating’s reflectivity [30].

## 3. Gratings Thermal Characterization

**Figure 2:**
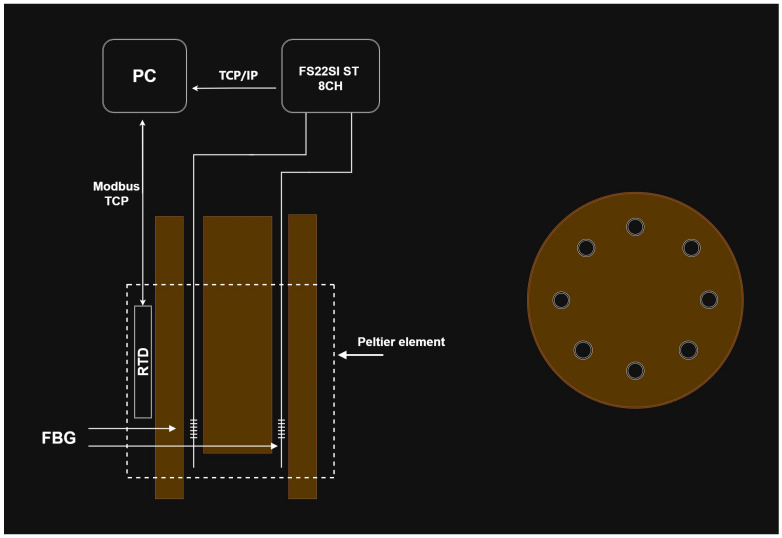
Schematic of the fiber Bragg grating temperature measurements calibration. The drawing on the right represents the cross section of the insert block (not in scale).

**Figure 3:**
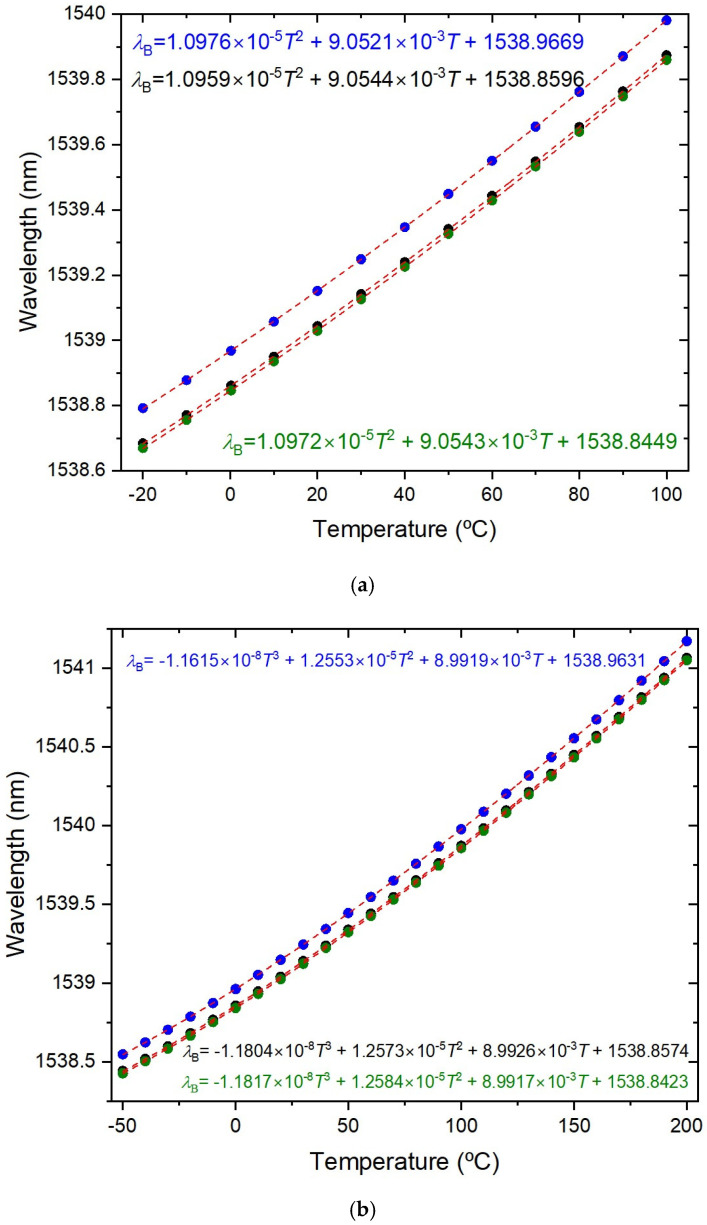
Bragg wavelength temperature dependence for the three UV-FBGs induced in the SMF-28 fiber: (**a**) four heating cycles −20–100 °C and (**b**) four heating cycles −50–200 °C (average values for each FBG).

**Figure 4:**
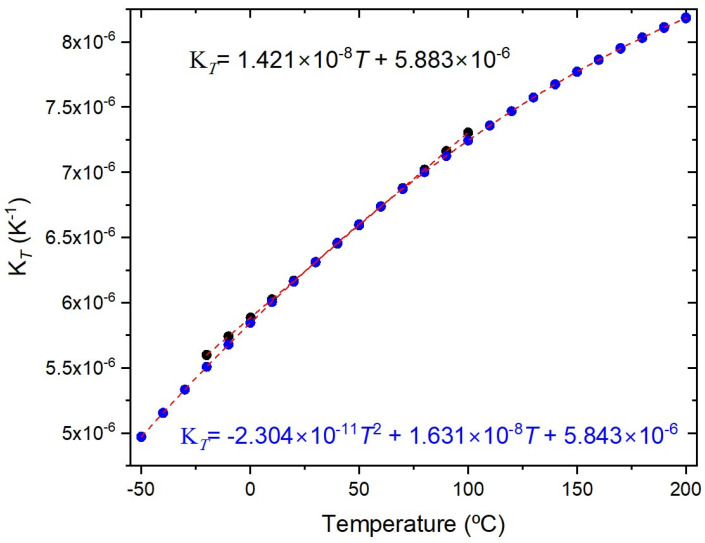
K*_T_* versus temperature (average values) considering the three FBGs for both temperature cycles.

The temperature characterization was performed using a Dry block-temperature calibrator from Sika, model type TP17200S, allowing measurements between −55 °C and 200 °C with 0.01 °C resolution and an accuracy of 0.2 °C. Gratings were mounted inside the insert blocks, keeping them stress-free. Bragg wavelengths were measured, after ~1 h stabilization, using an optical interrogator FS22SI BraggMeter ST (with eight channels), with an acquisition rate of 1 sample/s, which enables resolution/stability better than 0.5 pm/1 pm. Figure 2 shows that a PC collects the temperature measurements from the Dry block and the wavelength readings from the optical interrogator. Also shown is the position of the FBGs inside the insert block (eight holes). It should be stressed that each value communicated by Sika is an average of 20 temperature measurements (each registered 130 ms). The temperature range was from −20 °C up to 100 °C (cycle: 30 °C up to 90 °C, followed by 100 °C down to −20 °C, and finally −10 up to 30 °C, all in steps of 20 °C). At each temperature step, the Bragg wavelength was determined by averaging 2500 data points and the standard deviation was lower than 0.7 pm, with a typical value of 0.4 pm. The fitting to the experimental data followed the standard procedure of analyzing the coefficient of determination (R^2^~1), the residuals of the wavelength fitting and the residual sum of squares (RSSs) [24,28]. Therefore, the order of the polynomial fitting was determined by R^2^ having essentially a value of 1 (R^2^ ≥ 0.99999), and when the RSSs did not reduce substantially by performing a higher order fitting. A quadratic fitting was applied to the temperature dependence of the Bragg wavelength and it was observed that K*_T_* shifted linearly with temperature. Similarly, gratings inscribed in the SMF-28 fiber were submitted to four heating cycles from −50 °C up to 200 °C. A cubic fitting was applied to the temperature dependence of the Bragg wavelength and K*_T_* shifted quadratically with temperature. Figure 3 shows the temperature dependence of the Bragg wavelength for both temperature cycles. The comparison between the fitting of K*_T_* is presented in Figure 4, where it can be observed that a divergence occurs as one approaches the limits of the temperature interval [−20, 100] °C. This divergence can be explained by the fact that, at room temperature, K*_T_* = ((1/*n*_eff_)(d*n*_eff_/dT) + α_SiO2_) (the normalized thermo-optic coefficient and the thermal expansion coefficient, respectively) is essentially determined by the temperature behavior of the thermo-optic coefficient (d*n*_eff_/dT), a cubic temperature dependence [21,23,31,32,33,34,35]. The calibrations were repeated with all fiber gratings, as shown in Table 2, and the results are summarized in Table 3. It can be observed that K*_T_* increases with the fiber GeO_2_ content, being lower for the B/Ge fiber. The absolute values of K*_T_* for the SMF-28 fiber and Leoni SMF with an Ormocer coating differs from the ones previously measured [25]. Therefore, we performed a reanalysis of those experiments and the results will be discussed in the next section. The possible effect of the hydrogen-loading process or the grating coupling strength (reflectivity) on K*_T_* values was studied using gratings in the SMF-28e+ fiber. The results have shown that up to a reflectivity of 15% there is no impact on the determination of the normalized temperature sensitivity. Similar results were obtained for the other fibers, namely, a grating induced in the PS1500 (NA = 0.29) fiber with a reflectivity as high as 33% was tested, and gratings inscribed in a batch of the Leoni SMF with an Ormocer coating, having reflectivity ranging from 1% up to a saturated level, were also used.

## 4. Error Analysis Related to FBG Thermal Characterization

This section, besides its scientific content, also intends to be a pedagogical one in an era of unprecedent exponential increase in “insufficiently mature” published data. The initial thermal characterizations were performed using a customized oven, as described in [36] (although with shorter dimensions) and the thermal bath described in [25]. However, both heating apparatuses exhibited errors in the absolute temperature measurements. We realized that a 5 pm difference (or equivalently a temperature difference of 0.5 °C) on each temperature step led, after fitting the temperature dependence of the Bragg wavelength, to values of K*_T_* at room temperature (20 °C) that were expected for 29.4 °C, clearly being not sufficient for the precision required [23]. Therefore, all further characterizations were performed using the calibrated Dry block described in the previous section, and, using this new setup (Dry block + optical interrogator), we have investigated the potential source of errors in the determination of the temperature dependence of the Bragg wavelength, for which hundreds (above 750) of independent temperature measurements were performed over the last 18 months.

**Figure 5:**
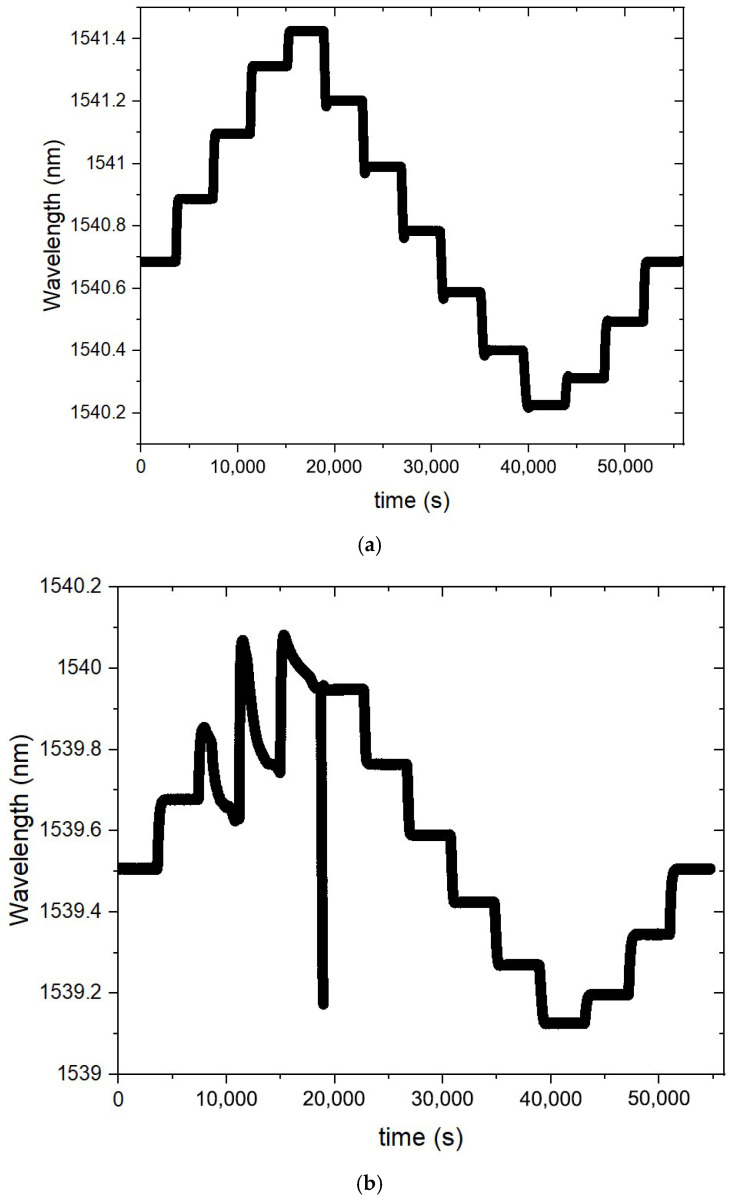

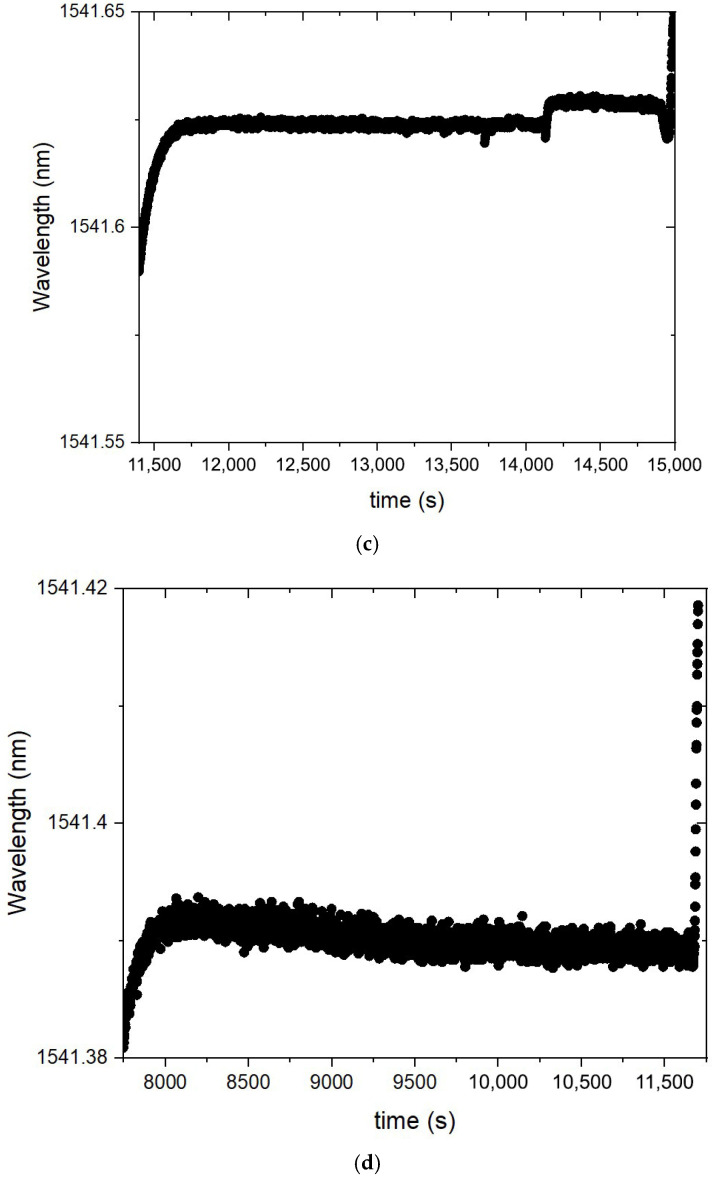
(**a**) Stable measurements corresponding to the raw data (wavelength) from the optical interrogator during a temperature cycle (from 30 °C up to 100 °C, down to −20 °C and up to 30 °C) for ~16 h; (**b**) instability at 70 °C, 90 °C and 100 °C steps; (**c**,**d**) instability at single temperature steps (initially checked by the larger values of standard deviation).

The potential effect of using different optical interrogators (comparison with a FS22SI with four channels) was analyzed. We have tested three FBGs (*λ*_B_~1543 nm) inscribed in the SM1500 (NA = 0.20) and another three FBGs (*λ*_B_~1541 nm) induced in the Leoni SMF fiber (with an Ormocer coating). Three heating cycles were performed in the temperature interval: −20–100 °C, using the eight channels unit and four more for the four channels unit, for a total of 34 tests. All K*_T_* values at 20 °C were in the interval ±0.004 K^−1^; therefore, both interrogators register the same values of Bragg wavelengths and, consequently, lead to the same K*_T_* values. We have identified another source of error related to a temperature gradient inside the calibration inserts (15.0 cm long) containing the FBG, which was estimated to be ~0.3 °C/cm outside the homogeneous temperature region. For instance, by using an array of two gratings inscribed in the SM1500 (NA = 0.30) fiber, separated by 6.0 cm, a difference in K*_T_* value of 0.006 K^−1^ was obtained, which corresponds to 0.5 °C. Therefore, one grating being 2.0 cm outside the uniform region yields a temperature gradient of 0.25 °C/cm. Values ranging from 0.25 up to 0.35 °C/cm were obtained by using different arrangements: gratings inserted at different depths (5 to 15 cm) and an array with five 4.5 mm gratings separated by ~10 mm. The Sika manual states that the homogeneous temperature zone is limited to 4.0 cm; therefore, the FBG should be well inside that region at the bottom of the insert. Since the temperature stability is very important to obtain good measurements, for each temperature step we analyzed the standard deviation, which was typically better than 0.4 pm, and we calculated the average of the resonance wavelength, typically using 2500 data points. When we closed the cycle, returning to the initial temperature, we also checked the resonance wavelength; the difference should be lower than 1 pm. Sometimes, the device loses stability, probably associated with condensation, which is observed in Figure 5b–d (wavelength vs. time), which also impacts the values obtained for K*_T_*. In such extreme cases, the measurements were discarded and only those exhibiting standard deviations lower than 1.0 pm were considered. It should be stressed that we have tried to minimize the occurrence of condensation inside the Dry block by increasing the temperature above 100 °C in the first step before decreasing to 30 °C, but the improvement is not always clear, nor is it uniform for all channels. The frequency of instability events seems to be associated with changes in temperature and humidity in the lab room throughout the year. As an example, it was difficult to reach stable values of temperature at −50 °C during summer and, therefore, we limited measurements down to −40 °C.

**Figure 6:**
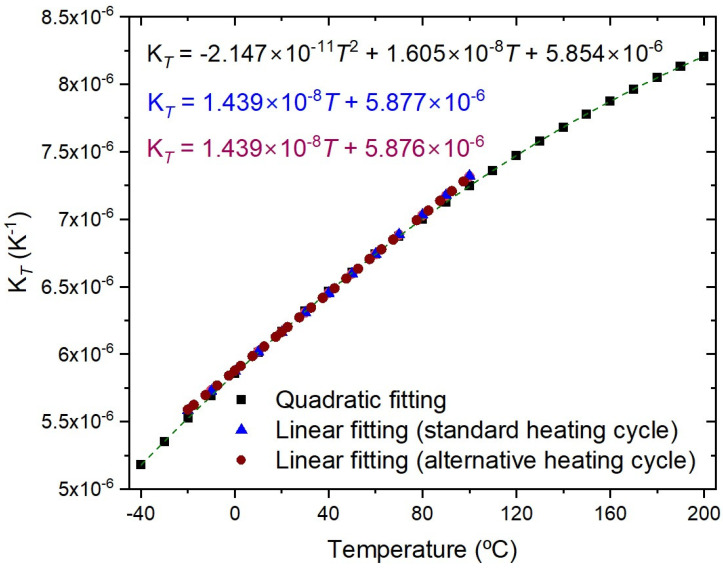
K*_T_* obtained for different temperature ranges and temperature steps.

When the above sources of error are controlled, we still have to deal with the curve fitting. A thorough study on the measurements’ repeatability was performed by using three gratings inscribed in the SMF-28 fiber. Four heating cycles (−20–100 °C) followed by another four heating cycles (−50–200 °C) were performed. Recently (6-month interval), five heating cycles (−40–200 °C) were also performed to compare reproducibility (measurements were limited to −40 °C, as mentioned above). For the former, a 2nd order polynomial fitting was applied to the temperature dependence of the Bragg wavelength, while a wider temperature range demands a higher polynomial fitting (Figure 3). As can be observed in Figure 4 and Figure 6, the normalized temperature sensitivity value is slightly lower for the latter. Nevertheless, a similar value is obtained when restricted to the same temperature range. It should be mentioned that K*_T_* values at 20 °C do not differ by more than 0.01 in each temperature range, totaling 39 independent measurements. We have also analyzed the influence of decreasing the temperature step, by performing three tests between −40 °C and 100 °C such that, when merged with previous tests, it results in temperature steps of about 2.5 °C. The K*_T_* values are similar to the ones obtained in previous tests (Linear-alternative in Figure 6). In general, gratings in other fibers show similar behavior.

**Figure 7:**
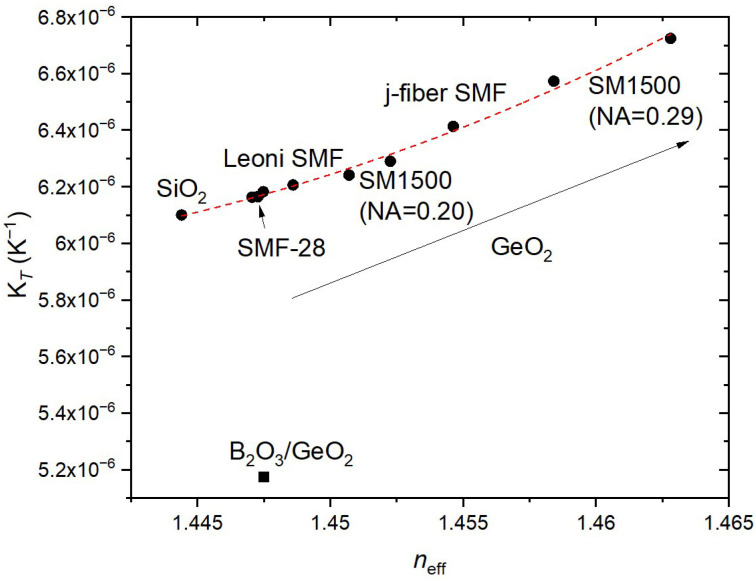
K*_T_* vs. *n*_eff_ @20 °C, obtained for various gratings inscribed in fibers with different germanium concentration. The red line is the fitting of the K*_T_* vs. *n*_eff_ (expression given in the text) as the GeO_2_ concentration increase (pointed by the arrow).

The last source of error, and one of the most important, is the homogeneity of the fiber itself, that is, the uniform distribution of the germanium concentration along the fiber core. As mentioned previously, we found values for fs-FBGs ranging from 6.175 × 10^−6^ K^−1^ up to 6.224 × 10^−6^ K^−1^, and from 6.153 × 10^−6^ K^−1^ up to 6.213 × 10^−6^ K^−1^ for UV-FBGs. The lack of uniformity was pointed out in the past during the fabrication of mechanically induced gratings in the SMF-28 fiber [37] and arc-induced gratings in the B/Ge co-doped fiber [20]. In this work, we have used the Leoni SMF fiber as a reference, since we had commercially available fs-FBGs on it, and, therefore, we could make comparisons between FBGs produced under different fabrication techniques and parameters, such as reflectivity. Initially we attributed the differences to the fs inscription [38], but, after a comparison of UV-FBGs produced in the SMF-28 fiber and in the Leoni SMF fiber using different setups ([25] and current work), we noticed that, while the ones inscribed in the SMF-28 fiber show similar values of K*_T_*, the same did not happen for FBGs induced in the Leoni SMF fiber. Moreover, recently we have inscribed FBGs in two new commercially available batches of SM1500 fiber (both NA = 0.20 and NA = 0.30), and, not only is the Bragg wavelength much higher (Δn_core_ = 1.555 × 10^−3^ and 4.390 × 10^−3^, which corresponds to an estimated increase in GeO_2_ concentration, Δ*n* = 1.464 × 10^−3^ × [GeO_2_(mol%)], of 1.1 and 3.0 mol%, respectively [23]), but K*_T_* is also higher for FBGs induced in both fibers: from 6.238 × 10^−6^ K^−1^ to 6.290 × 10^−6^ K^−1^ and from 6.574 × 10^−6^ K^−1^ to 6.724 × 10^−6^ K^−1^, respectively. Therefore, we have used 18 m of the Leoni SMF fiber containing two fs-FBGs (~19%; (6.182 ± 0.001) × 10^−6^ K^−1^) to inscribe UV-FBGs with different reflectivity (<1%, 18.9%, 98.8% and saturated), using both setups and all values of K*_T_* falling in the range (6.186 ± 0.006) × 10^−6^ K^−1^. It should be highlighted that peak detection for the high reflectivity gratings is also a demanding task (and the values of R^2^ of the fitting are lower for these gratings). Therefore, since we cannot guarantee that there is a uniform distribution of germanium in the core of the 18 m length of the Leoni SMF fiber, under the experimental conditions, we may conclude that K*_T_* is similar for fs-FBGs and UV-FBGs and that the coupling strength has a negligible influence on the determination of K*_T_*. For the sake of illustration, and since we do not know the fiber parameters in order to determine germanium concentration, we present K*_T_* as a function of *n*_eff_ in Figure 7. We have also included the K*_T_* value (K*_T_* = 1.418 × 10^−8^ *T* + 6.130 × 10^−6^ K^−1^; 6.413 × 10^−6^ K^−1^ @20 °C) obtained using three gratings (*λ*_B_~1540, 1547 and 1554 nm) inscribed in another high Ge-doped fiber (j-fiber SMF: NA = 0.26 and MFD = 5.5 ± 0.4). It is clearly shown that K*_T_* increases with the increase in GeO_2_ concentration and decreases with B_2_O_3_ concentration. For FBGs inscribed in Ge-doped fibers, the expression K*_T_* = 4.472 × 10^−4^ *n_eff_*^2^ − 1.270 × 10^−3^ *n_eff_* + 9.075 × 10^−4^ (red line in Figure 7) can be used to estimate the K*_T_* value at 20 °C, as a function of the effective refractive index, with a typical error of 1 × 10^−8^ K^−1^ being the worst-case scenario, for highly Ge-doped fibers, of 7 × 10^−8^ K^−1^ (a “K*_T_*” value of 19.84 × 10^−6^ K^−1^, for bulk germanium glass was also considered [23]; shown in Supplementary Materials).

## 5. Prediction of the Temperature Dependence of the Bragg Wavelength

Assuming that we know *K*(*T*) for one UV-FBG and *λ*(*T*_0_) for a second FBG inscribed in the same fiber, we can determine *λ*(*T*) for the latter, through the integration of *K*(*T*) and by applying a 3rd order Taylor expansion to the exponential function. Thus,

(1) $$K\left(T\right)=\frac{1}{\lambda }\frac{d\lambda }{dT}=K_{2}T^{2}+K_{1}T+K_{0},$$

or

(2) $$K\left(T\right)=K_{2}{\left(T-T_{0}\right)}^{2}+\left(K_{1}+2K_{2}T_{0}\right)\left(T-T_{0}\right)+K(T_{0}),$$

and therefore,

(3) $$\begin{array}{l} \lambda \left(T\right)=\lambda \left(T_{0}\right)*[1+K\left(T_{0}\right)*\left(T-T_{0}\right)+\left(\frac{K_{1}+2K_{2}T_{0}+{K\left(T_{0}\right)}^{2}}{2}\right)*{\left(T-T_{0}\right)}^{2}+\\ \;\;\;\;\;\;\left(\frac{K_{2}}{3}+\frac{{K\left(T_{0}\right)}^{3}}{6}+\frac{\left(K_{1}+2K_{2}T_{0}\right)*K(T_{0})}{2}\right)*{\left(T-T_{0}\right)}^{3}], \end{array}$$

and

(4) $$\begin{array}{l} \frac{d\lambda (T)}{dT}=\lambda \left(T_{0}\right)*[K\left(T_{0}\right)+\left(K_{1}+2K_{2}T_{0}+{K\left(T_{0}\right)}^{2}\right)*\left(T-T_{0}\right)+\\ \;\;\;\;\;\left(K_{2}+\frac{{K\left(T_{0}\right)}^{3}+3\left(K_{1}+2K_{2}T_{0}\right)*K(T_{0})}{2}\right)*{\left(T-T_{0}\right)}^{2}], \end{array}$$

where *K_i_* (*i* = 0, 1, 2) are coefficients. From the previous sections, it is clear that a quadratic polynomial fits, with high correlation, the temperature dependence of the Bragg wavelength in the [−20, 100] °C temperature range. Furthermore, increasing the polynomial order would result in a negligible reduction in the RSSs. Thus, a linear behavior for *K_T_* is adequate, and therefore, *K*_2_ = 0. We demonstrate the applicability by using five gratings written in the SM1500 (NA = 0.20) fiber with a reflectivity of about 0.5%. Table 4 summarizes the average values of *K_T_* at 20 °C for four tests in the [−40, 200] temperature range, limited to the [−20, 100] °C, and also three tests in the [−20, 97.5] °C, in steps of 10 °C, but at different temperature plateaus. For each grating, the absolute error of *K_T_* is within 0.002 K^−1^. Therefore, there is a slight dependence of *K_T_* on wavelength (in a range that exceeds 50 nm) that was averaged in the subsequent calculations. Thus, by fitting all experimental data, for the first temperature range, we obtained *K_T_* = −2.197 × 10^−11^*T*^2^ + 1.669 × 10^−8^*T* + 5.919 × 10^−6^, for the second, we obtained *K_T_* = 1.500 × 10^−8^*T* + 5.941 × 10^−6^ and for the last, we obtained *K_T_* = 1.512 × 10^−8^*T* + 5.936 × 10^−6^ (R^2^ = 0.9999). Table 5 summarizes the results obtained by comparison of the Bragg wavelengths and temperature sensitivity (d*λ*/d*T*) values from direct calibration (d*λ*__dir2_/d*T* and d*λ*__dir3_/d*T*, for quadratic and cubic fitting, respectively) and by following Equations (1)–(4), where we have assumed *K_T_* through the fitting of all experimental data of the five FBGs (*λ*__int2_ and d*λ*__dint2_/d*T*, for quadratic fitting and *λ*__int3_ and d*λ*__dint3_/d*T*, for cubic fitting). In each temperature range, the difference in wavelength is lower than 3 pm and the calculated temperature sensitivity differs by ~0.01 pm/°C. It should be noted that the differences in the temperature sensitivity near the limits of the temperature range (0.1 pm/°C) are essentially due to the dependence of *K_T_* on temperature (which goes from quadratic to cubic dependence for larger temperature interval), despite its slight dependence on wavelength for the five FBGs inscribed in this fiber with high GeO_2_ dopant concentration (as observed in Table 4 and with impact on the fitting: R^2^ = 0.9999). Concerning the [−20, 97.5] °C temperature range, the values of *K_T_* at 20 °C have lower dispersion (Table 4), and similar results were obtained when applying the method to estimate the wavelength and temperature sensitivity of the “unknown” grating.

A similar analysis was performed using five gratings inscribed in the SMF-28 fiber and K*_T_* was calculated in the −20–100 °C temperature range. Table 6 summarizes the average values of d*λ*/d*T* and K*_T_* for four independent measurements. Note that the dependence of the temperature sensitivity on wavelength is well-expressed in Equation (4). All results are within 6.165 ± 0.004 K^−1^, being more stable than the ones obtained for the previous gratings inscribed in the SM1500 fiber. Table 7 shows the results of considering the methodology by using K*_T_* = 1.424 × 10^−8^*T* + 5.883 × 10^−6^ obtained for all FBGs and by applying it to the FBGs at 1561.6 nm. The largest difference, 2.4 pm, occurs at −20 °C, but the sensitivity is always within 0.01 pm/°C. It should be stressed that we analyzed the independence of wavelength using gratings inscribed in other fibers and the results were even more constant, within 0.001, although for shorter wavelength ranges: SM1500_NA = 0.30 (two FBGs; 10 nm), j-fiber SMF (three FBGs; 14 nm) and Leoni SMF (five gratings; 27 nm).

**Figure 8:**
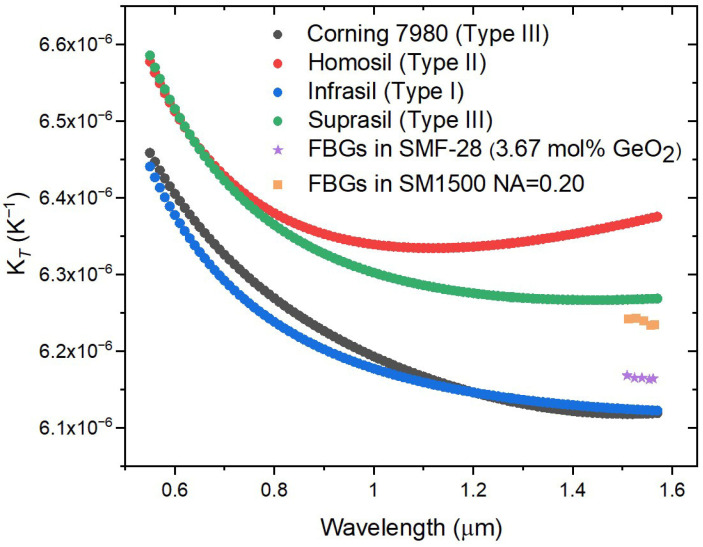
“K*_T_*” for four silica glasses [21,22,39,40,41] and K*_T_* for UV-FBGs inscribed in the SMF-28 Corning fiber and in FiberCore SM1500 fiber (NA = 0.20).

Figure 8 shows the wavelength dependence of “K*_T_*” for four silica glasses and K*_T_* for five FBGs inscribed in the SMF-28 Corning fiber and in the SM1500 fiber (NA = 0.20). As can be observed, the wavelength dependence of K*_T_* is negligible for the FBGs in the SMF-28 fiber (and also resembles the “K*_T_*” behavior of the Corning glass). On the other hand, K*_T_* seems to have a slight dependence on wavelength for FBGs in the SM1500 fiber.

## 6. Discussion of the Results

The results obtained depend on the stability and resolution of the devices used. The optical interrogator has a sub-picometer resolution (wavelength is determined with eight digits) and the Dry block has a temperature resolution of 0.01 °C (four digits). However, the temperature registered every second is an average of 20 measurements and, therefore, we have assumed five digits for intermediate calculations. On the other hand, it is important to recognize that, sometimes, we are comparing results for the same gratings obtained with intervals of several months, and not only do the environmental conditions change, but both devices require calibration at least every year. In fact, it should be highlighted that the requirement for calibration was the main cause of the recent instability measurements discussed previously, since the replacement for another optical interrogator surpasses that event. Despite the good results obtained overall, there is room for improvement, and not only will the Dry-block be placed in a space with a controlled environment (temperature and humidity), but also an external calibrated temperature sensor with higher resolution will be inserted in a hole to be drilled in the center of the insert block containing the FBG (Figure 2), in order to guarantee higher temperature accuracy and resolution.

Concerning the polynomial fitting to the temperature dependence of the Bragg wavelength, from the theoretical point of view, the higher the order of the polynomial, the better the fitting, at least up to the fourth order, since, as discussed, the thermo-optic coefficient follows a cubic temperature dependence. However, depending on the temperature interval, and based on the coefficient of determination, residuals and RSSs, we assumed for the Bragg wavelength a quadratic and a cubic dependence for the temperature range −20–100 °C and −50–200 °C, respectively. In general, for each temperature step, we used an average of 2500 data points and the standard deviation was much lower than 1 pm (error bars in the figures would be smaller than the dimensions of the data points). Typically, for FBGs inscribed in the SMF-28 fiber, the error in K*_T_* @20 °C is of the order of 2 × 10^−9^ K^−1^ when considering different gratings but the same wavelength; it duplicates by taking into account different wavelengths, and including the fitting and tests over time, it is 5 × 10^−9^ K^−1^. This value may duplicate for FBGs inscribed in high Ge-doped fibers, as observed in the previous section.

Different fibers were used in this study in order to measure the impact of GeO_2_ concentration (or, at least, of *n*_eff_) on K*_T_* values, and also to understand if the potential dependence of K*_T_* on wavelength would differ from the “K*_T_*” behavior of bulk SiO_2_ glass, as the GeO_2_ concentration increases. Apparently, some dependence may exist for high Ge-doped fibers, but care should be taken, since these fibers are more temperature-sensitive, and, therefore, more prone to temperature measurement errors. A comparison of the K*_T_* values obtained for FBGs inscribed in the SMF-28 fiber (probably the most well-known fiber and the one exhibiting more stable parameters over time) is presented in Table 8. As can be seen, the value obtained in this work has higher accuracy and compares well with the value determined by Flockhart et al. [24], where they have also used a quadratic fitting, and with the one achieved by Imas et al. [27], taking into account that they have used a linear fitting for a temperature interval above 20 °C. Moreover, the value of K*_T_* = 6.165 × 10^−6^ K^−1^, at 20 °C, obtained accurately for FBGs inscribed in the SMF-28 fiber, allowed us to recalculate, following the methodology presented in [23], the thermo-optic coefficient of bulk germanium glass to be 19.4 × 10^−6^ K^−1^, being, therefore, in excellent agreement with the published literature [42]. Finally, the approach that we presented in the preceding section to estimate the wavelength dependence of the “unknown” FBGs by considering the K*_T_* from another grating also reinforces the accuracy of the experimental results.

In [43], a much higher value of K*_T_* = (7.19 ± 0.16) × 10^−6^ K^−1^ was also presented for an FBG inscribed in a SM1500 fiber (the conditions were the same as those described in the table above). K*_T_* values of 5.86 × 10^−6^ K^−1^ and 5.95 × 10^−6^ K^−1^ (standard deviation for temperature and wavelength were 0.6–0.9 °C and 5–8 pm, respectively) were also obtained for FBGs inscribed in a PS1250/1500 fiber [44]. In this case, a quadratic fitting was used from 22 °C up to 250 °C and, therefore, the uncertainty is very high to extrapolate the correct value at 20 °C, these values also being much higher than the one obtained in this work.

## 7. Conclusions

We have shown that the temperature dependence of the normalized temperature sensitivity, K*_T_*, was found to be quadratic in the −50–200 °C range while it is linear for the −20–100 °C range. K*_T_* values at 20 °C range from 5.176 × 10^−6^ K^−1^, for a B/Ge co-doped fiber up to 6.724 × 10^−6^ K^−1^, for a highly Ge-doped fiber. We have demonstrated that K*_T_* does not depend on the hydrogen-loading process, nor on the gratings coupling strength, and that is independent of wavelength in the 1500–1600 nm range. Furthermore, gratings produced by femtosecond-laser radiation and UV-laser radiation exhibit comparable values of K*_T_*. The K*_T_* expressions for FBGs inscribed in silica fibers with distinct concentrations of GeO_2_ were given. A K*_T_* = 6.165 × 10^−6^ K^−1^, at 20 °C, was accurately obtained for FBGs inscribed in the SMF-28 fiber, which allowed us to calculate the thermo-optic coefficient of bulk germanium glass to be 19.4 × 10^−6^ K^−1^. A discussion of the potential sources of error on K*_T_* determination was presented. We have introduced a methodology that allows the accurate determination of the temperature dependence of the Bragg wavelength of any grating, as far as one has knowledge of K*_T_* for a single grating inscribed in the same fiber. This methodology is very useful in research labs and computer simulations, being a step further towards the determination of the thermo-optic coefficient of ternary glasses, such as the B/Ge co-doped silica glass fiber (FiberCore PS125/1500). Therefore, by also having knowledge of the fiber parameters (namely, the refractive index profile), we expect to estimate the temperature dependence of the thermo-optic coefficient of such glass and accurately determine the dispersion turning points of arc-induced gratings at cryogenic temperatures. This would be the ultimate corner to be turned towards the development of high-sensitivity cryogenic temperature sensors based on arc-induced gratings.

## Figures and Tables

**Table 1:** Properties of the fibers used in this work.

Fibers	NA	MFD (μm)
Corning SMF-28	0.14	10.4 ± 0.8
Corning SMF-28e+	0.14	10.4 ± 0.5
Leoni SMF (w/Ormocer coating)	0.14	8.8 ± 0.5
FiberCore SM1500 (w/high GeO_2_-dopant)	0.20	4.3 ± 0.2
FiberCore SM1500 (w/high GeO_2_-dopant)	0.30	6.4 ± 0.4
FiberCore PS1250/1500 (B/Ge co-dopants)	0.13	9.7 ± 0.9

**Table 2:** Gratings reflectivity and UV fluence for the gratings inscribed in six different fibers.

SMF-28e+ Pristine		SMF-28e+ H_2_ Loaded		SM1500 NA = 0.30		PS1250/1500, NA = 0.13	
R (%)	F (J/mm^2^)	R (%)	F (J/mm^2^)	R (%)	F (J/mm^2^)	R (%)	F (J/mm^2^)
7.3	48.8	15	3.30	33	0.857	13	2.308
1.2	9.76	4	1.32	12	0.429	4	0.923
0.36	4.62	1.3	0.264	2.8	0.00536	1.5	0.154
0.07	0.923	0.095	0.0528	0.7	0.00165	0.26	0.0264
SMF-28		Leoni SMF (w/Ormocer coating)		SM1500 NA = 0.20			
R (%)	F (J/mm^2^)	R (%)	F (J/mm^2^)	R (%)	F (J/mm^2^)		
1.6	9.89	1.48	9.89	10	0.923		
0.46	4.94	0.55	4.94	1.0	0.0923		
0.09	0. 989	0.13	0.989	0.51	0.00385		

**Table 3:** KT expressions for FBGs inscribed in different fibers, in the temperature range [−20, 100] °C.

Fiber	K*_T_* (K^−1^) (*T* Is in °C in Equations Below)	K*_T_* @20 °C
FiberCore PS1250/1500	1.562 × 10^−8^ *T* + 4.864 × 10^−6^	5.176 × 10^−6^
Corning SMF-28 [−50, 200] °C	−2.304 × 10^−11^ *T*^2^ + 1.631 × 10^−8^ *T* + 5.843 × 10^−6^	6.166 × 10^−6^
Corning SMF-28	1.431 × 10^−8^ *T* + 5.879 × 10^−6^	6.165 × 10^−6^
Corning SMF-28e+	1.435 × 10^−8^ *T* + 5.878 × 10^−6^	6.165 × 10^−6^
Leoni SMF (w/Ormocer coating)	1.452 × 10^−8^ *T* + 5.896 × 10^−6^	6.186 × 10^−6^
FiberCore SM1500 NA = 0.20	1.512 × 10^−8^ *T* + 5.936 × 10^−6^	6.238 × 10^−6^
FiberCore SM1500 NA = 0.30	1.589 × 10^−8^ *T* + 6.256 × 10^−6^	6.574 × 10^−6^

**Table 4:** K*_T_* values at 20 °C obtained using five FBGs induced in the SM1500 (NA_0.20).

	*λ*_B_ (nm)	1512.7	1527.7	1542.8	1557.7	1565.1
<K*_T_* (K^−1^)>						
[−40, 200] (°C)		6.248 × 10^−6^	6.250 × 10^−6^	6.246 × 10^−6^	6.239 × 10^−6^	6.239 × 10^−6^
[−20, 100] (°C)		6.244 × 10^−6^	6.249 × 10^−6^	6.242 × 10^−6^	6.237 × 10^−6^	6.235 × 10^−6^
[−20, 97.5] (°C)		6.242 × 10^−6^	6.243 × 10^−6^	6.239 × 10^−6^	6.233 × 10^−6^	6.234 × 10^−6^

**Table 5:** Calculated Bragg wavelength and temperature sensitivity for the “unknown” grating.

*T* (°C)	*<λ>* (nm)	*λ*__int2_ (nm)	*λ*__int3_ (nm)	d*λ*__int2_/d*T* (pm/°C)	d*λ*__dir2_/d*T* (pm/°C)	d*λ*__int3_/d*T* (pm/°C)	d*λ*__dir3_/d*T* (pm/°C)
−39.94	1527.2213	-	1527.2195	-	-	7.969	7.977
−20	1527.3844	1527.3825	1527.3840	8.616	8.628	8.519	8.527
0.1	1527.5608	1527.5603	1527.5605	9.077	9.089	9.046	9.054
20	1527.7455	1527.7455	1527.7455	9.535	9.546	9.541	9.548
100	1528.5831	1528.5819	1528.5806	11.374	11.383	11.266	11.269
200	1529.7931	-	1529.7905	-	-	12.821	12.814

**Table 6:** d*λ*/d*T* and K*_T_* values at 20 °C obtained using five FBGs inscribed in the SMF-28 fiber and Bragg wavelengths fitted in the [−20, 100] °C temperature range.

	*λ*_B_ (nm)	1509.2	1524.2	1539.2	1554.1	1561.6
	
d*λ*/d*T* (pm/°C)		9.309	9.401	9.489	9.578	9.626
K*_T_* (K^−1^)		6.168 × 10^−6^	6.165 × 10^−6^	6.165 × 10^−6^	6.163 × 10^−6^	6.164 × 10^−6^

**Table 7:** Calculated Bragg wavelength and temperature sensitivity for the “unknown” grating.

*T* (°C)	*<λ>* (nm)	*λ*__int2_ (nm)	d*λ*__int2_/d*T* (pm/°C)	d*λ*__dir2_/d*T* (pm/°C)
−20	1561.2157	1561.2133	8.740	8.736
0.1	1561.3939	1561.3934	9.188	9.183
20	1561.5807	1561.5807	9.632	9.626
100	1562.4223	1562.4226	11.417	11.407

**Table 8:** KT values obtained for FBGs inscribed in the SMF-28/SMF-28e+ fibers.

K*_T_* (×10^−6^ K^−1^)	Standard Error (×10^−6^ K^−1^)	Temperature Interval (°C)	Fitting	Reference
6.165	0.005	20	quadratic	This work
6.25	0.02	10–50	linear	[27]
6.50	0.05	3–35	linear	[30]
6.14	0.05	20	quadratic	[24]
6.65	0.06	30–80	linear	[43]

## Data Availability

The data segments can be obtained by contacting the corresponding author.

## Supplementary Materials

The following supporting information can be downloaded at: https://www.mdpi.com/article/10.3390/s26020435/s1, Figure S1: KT vs. neff from SiO_2_ up to GeO_2_.
