# Supplementary figures and images for: Accurate Determination of the Temperature Sensitivity of UV-Induced Fiber Bragg Gratings
Source: Sensors (Basel). 2026 Jan 9;26(2):435. doi: 10.3390/s26020435 (PMC12846186; doi:10.3390/s26020435)

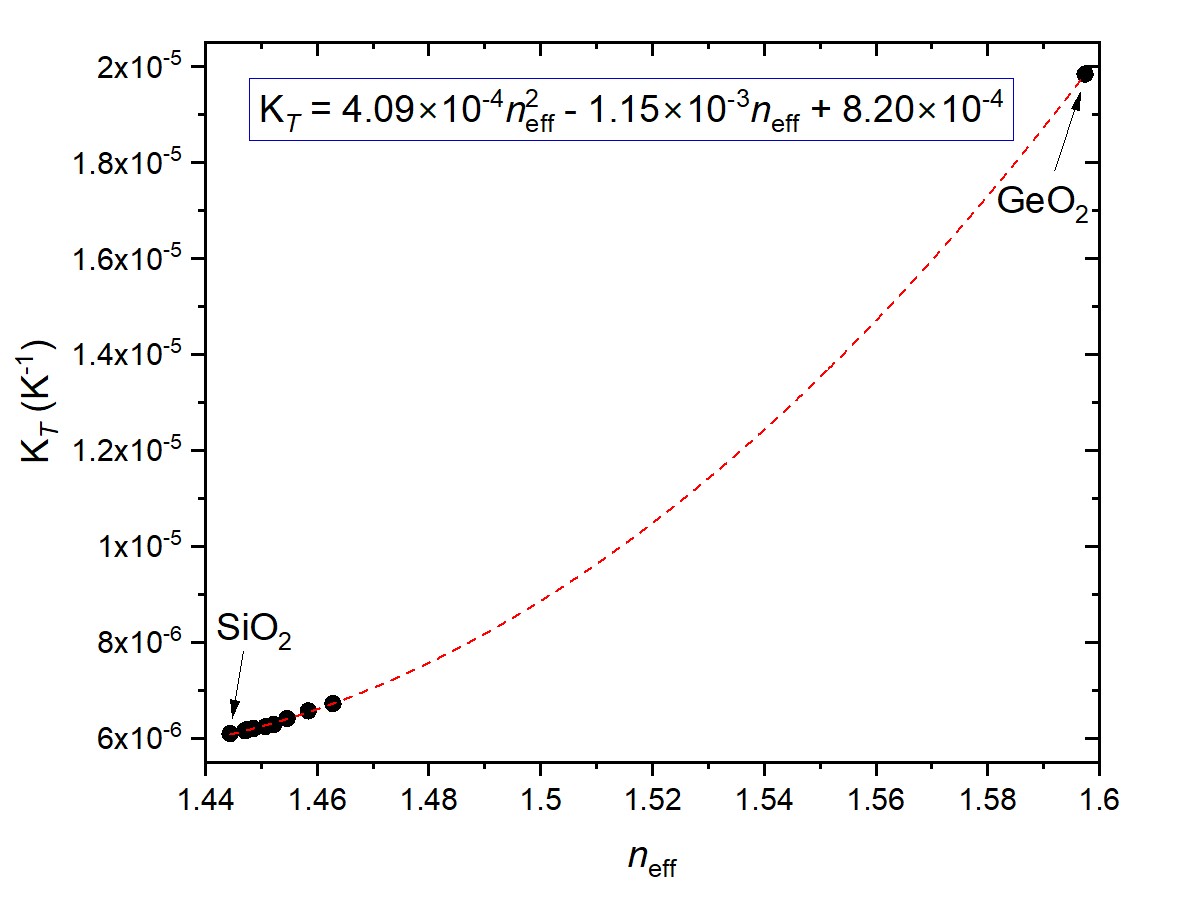

Supplement: Supplementary file 1 [file sensors-26-00435-s001.zip › Supplementary Figure S1.jpg]
